# Retraction: Molecular Characterization and Antibiotic Susceptibility Pattern of Bacterial Strains Isolated From Wound of Patients With Diabetes

**DOI:** 10.7759/cureus.r138

**Published:** 2024-03-13

**Authors:** Eemaz Nathaniel, Jibran Ikram, Aimen James, Bakhtawar Obaid, Ayesha Zahid, Zeeshan Ahmed, Dilawar K Wazir, Qazi Muhammad Farooq Wahab, Giustino Varrassi, Satesh Kumar, Mahima Khatri

**Affiliations:** 1 Research, Rehman Medical Institute, Peshawar, PAK; 2 Orthopaedics and Trauma, Rehman Medical Institute, Peshawar, PAK; 3 Dermatology, Rehman Medical Institute, Peshawar, PAK; 4 Internal Medicine, Rehman Medical Institute, Peshawar, PAK; 5 Surgery, Rehman Medical Institute, Peshawar, PAK; 6 Medicine, Hayatabad Medical Complex Peshawar, Peshawar, PAK; 7 Microbiology, University of Peshawar, Peshawar, PAK; 8 Medicine, Khyber Teaching Hospital, Peshawar, PAK; 9 Pain Medicine, Paolo Procacci Foundation, Rome, ITA; 10 Medicine and Surgery, Shaheed Mohtarma Benazir Bhutto Medical College, Karachi, PAK; 11 Medicine and Surgery, Dow University of Health Sciences, Karachi, PAK

This article has been retracted by the Editor-in-Chief due to plagiarism. This article was previously published in Endocrine and Metabolic Science: https://www.sciencedirect.com/science/article/pii/S2666396123000134?via%3Dihub. An investigation was conducted by Hayatabad Medical Complex, which confirmed that the article contents were shared with others who then submitted and published the same study in Cureus while fraudulently claiming authorship. As a result, this article has been retracted.

